# Does a decision aid improve informed choice in mammography screening? Study protocol for a randomized controlled trial

**DOI:** 10.1186/s12905-015-0210-5

**Published:** 2015-07-22

**Authors:** Maren Reder, Petra Kolip

**Affiliations:** Bielefeld University, School of Public Health, Department of Prevention and Health Promotion, Universitätsstraße 25, Bielefeld, 33615 Germany

**Keywords:** Mammography screening, Informed choice, Decision aid, Randomized controlled trial

## Abstract

**Background:**

When invited for the first time at age 50, most women in Germany have to decide whether they wish to participate in the German mammography screening programme. For ethical reasons, screening decisions should be informed choices, but this is rarely the case with mammography screening. Decision aids are interventions with the potential to support informed choice by improving the following factors: knowledge, clarity of personal attitude, and implementation of an intention. Currently, no systematically evaluated decision aid exists for the German mammography screening programme. Therefore, the objective of this randomized controlled trial is to assess the effectiveness of a decision aid for first-time mammography screening programme invitees.

**Methods/Design:**

We have developed a decision aid for women invited to the mammography screening programme for the first time based on the criteria of the International Patient Decision Aids Standards Collaboration. The effectiveness of the decision aid will be evaluated in a randomized controlled trial with a 3-month follow-up. We will invite 7400 women aged 50 years from the district of Westfalen-Lippe, Germany, to participate. This sample will be drawn from registration office data. The primary outcome will be informed choice. The secondary outcomes will be the components of informed choice (knowledge, attitude, decision/implementation). Decisional conflict, decision regret, eHealth literacy, health behaviours, perceived behavioural control, subjective norms, invitation status, and demographic variables will be assessed. Data will be collected online at baseline, post-intervention, and at the 3-month follow-up. Participants will be randomized to receive either the decision aid or usual care (invitation and standard leaflet of the mammography screening programme).

**Discussion:**

This paper describes the evaluation of a decision aid for the German mammography screening programme in a randomized controlled trial. If the decision aid proves to be an effective tool to enhance the rate of informed choice, it will be made accessible to the public and the use of this decision aid for first-time invitees will be recommended. The long-term effect could be an improvement in informed choices in women invited to the mammography screening programme.

**Trial registration:**

German Clinical Trials Register DRKS00005176.

**Electronic supplementary material:**

The online version of this article (doi:10.1186/s12905-015-0210-5) contains supplementary material, which is available to authorized users.

## Background

Informed choice has become an important public health issue [1]. In the face of growing discussion about the usefulness of mammography screening [2], there is an increased need to involve women in the screening decision. For ethical reasons, their choices should be informed [3]. Despite a lack of evidence for the all-cause mortality benefit of mammography screening [4], all women in Germany between the ages of 50 and 69 are invited every 2 years to the mammography screening programme (MSP). The invitation comes with a pre-specified appointment. However, each recipient can choose whether she wishes to participate. This decision is made in the context of scientific uncertainty [5] about the extent of benefits and harms of mammography screening. Nevertheless, the accompanying information provided by the inviting organization makes it appear that choosing to attend is a more appropriate decision, thus removing uncertainty from the message. Research shows that an informed choice is not achieved in a large proportion of mammography screening decisions: Mathieu et al. arrived at the figure of 48 % informed choices [6]; van Agt et al. found 88 % [7]. Indeed, the term “uninformed compliance” [8] has been coined to describe participation in this screening. Uninformed compliance is a major public health problem in the German MSP, since many women in the target group of the MSP have unrealistic expectations regarding its potential benefits [9]. Weighing benefits against harms is a value judgment, thus no correct answer can be determined [10] making this decision preference sensitive.

To identify solutions towards increasing the proportion of women making informed choices, the dimensions of this construct must be specified. A decision is classified as informed if the decision maker has good knowledge of the situation as well as an attitude congruent with the choice, and then implements the decision [11]. Therefore, to enable an informed choice, women invited to the MSP need to be informed about the existence and probability of positive and negative outcomes and given the opportunity to clarify the meanings of those outcomes for them personally. If these two conditions are met, implementation of a decision is also more likely.

The question then is how to meet these conditions. One innovative approach to addressing the issue of uninformed compliance is the use of decision aids (DAs). It is in preference sensitive decisions that a DA is most useful though the level of detail that should be used in DAs is controversial [12].

There is ongoing discussion about how useful quantitative information in DAs is for the deciding person [13]. From an informed choice perspective, not only the existence of a benefit or harm but also its probability is important for the decision [14]. Otherwise it is not possible to weigh the different outcomes against each other.

To assess concordance in decision making, several options exist. Sepucha and Fowler [15] recommend a simple match using decision (in form of a single preference question) and implementation; this leaves out attitude (which would require several questions on the different outcomes of an option). While preference already includes a decisional element, attitude is more distal targeting the different outcomes an option has. In this study, we therefore use attitude and knowledge scores to determine decision quality.

DAs are tools that help individuals choose between “two or more medically-appropriate healthcare options” [16] (p. 2). Research has shown that DAs are a promising strategy to increase the rate of informed choices in mammography screening [6]. To date, however, few studies have examined the effect of DAs on informed choice in such screening.

For the established MSP in Germany, there are no systematically evaluated DAs. To our knowledge, the only DA that exists was evaluated with a convenience sample of n=152 in a cross-sectional study design [17]. It does not meet International Patient Decision Aids Standards (IPDAS) to a high degree. Common shortcomings of studies in other mammography screening contexts are that they analyse only the immediate effects of DAs and do not follow up on the long-term effects on knowledge and decisional conflict [18]. Additionally, they do not target the age group for which mammography screening is intended [6, 19] or assess only high-risk populations [20, 21]. Therefore, it is unclear whether a DA would be beneficial for the German MSP or what long-term effects it would have. To meet this lack of a suitable DA, we developed a DA for first-time MSP invitees.

The present study aims to assess the effect of an interactive online DA on informed choice in a randomized controlled trial using a representative sample. The primary objective is to evaluate whether the DA results in more women making an informed choice. The secondary objectives are to evaluate whether the DA (1) improves knowledge of the MSP, (2) clarifies attitudes on the MSP, (3) changes intentions to participate in the MSP, (4) is affected by background factors (e.g. education level) in its influence on the primary and secondary outcomes, (5) lowers decisional conflicts, and (6) differs in its long-term effects depending on the outcome group of the screening (true positive, false positive, negative).

## Methods/Design

### Design

**Fig. 1:**
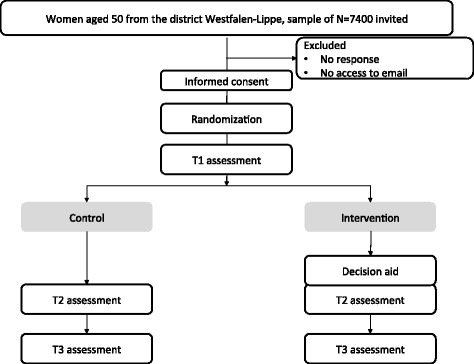
Trial flow diagram

This study is a non-blinded two-arm randomized controlled trial, since it was obvious to the participants whether they received just questionnaires or also an online decision aid. The participating women will be randomized to either (1) the DA (intervention group) or (2) usual care (control group). The random allocation will be on a 1:1 ratio basis. This study will be conducted in Wesfalen-Lippe, Germany. The usual care for women aged 50 in Germany involves an invitation from the MSP accompanied by an information leaflet (see [22]). Therefore, both study groups will receive these standard materials. The online assessments will be conducted at baseline (T1), at post-intervention (T2), and at a 3-month follow-up (T3) (Fig. 1).

### Ethical approval

Ethical approval was obtained from the Ethics Commission of the Medical Association Westfalen-Lippe and the Medical Faculty of the University of Münster. Data handling will conform to the data protection legislation of the federal state of North Rhine-Westphalia. We have also obtained approval from the data protection officer at Bielefeld University.

This research project is independent of the MSP. Invited women will be informed about the content, purpose, and procedure of the study, and we will obtain their written informed consent before proceeding. Personal data will be stored separately from research data. The first, second, and third assessments (T1, T2, and T3) can be linked to each other by means of a self-generated code consisting of the following elements: day of the month the mother was born, first letter of the first name of the mother, day of the month the father was born, first letter of the first name of the father, and first letter of own place of birth. This way the questionnaires cannot be associated with any personal data. All personal data will be deleted after completion of the study. Participation in the study will be voluntary and the participants may revoke consent at any time.

### Participants’ eligibility

Women aged 50 will be eligible for this study because they are invited to the MSP for the first time. Women who have ever been diagnosed with breast cancer will be excluded since they are not targeted by the MSP. Previous mammograms for diagnostic or screening purposes will not influence eligibility.

### Recruitment

The sample will be representative of the above-described study population in the district of Westfalen-Lippe. The data will stem from the registration offices of this district, which have consented to their data being used in this study. The sample will consist of women with birth months of March to May 1964. We will make the following random selection from our data pool: 2000 women with the birth month of March, 2000 women with the birth month of April, and 3400 women with the birth month of May. The sampling will take place (drawing without replacement) after the sampling of another study—Informed Choice of German and Turkish Women for Participation in the MSP (InEMa) [23]. All women with a positive name algorithm for a Turkish migration background have been assigned to the InEMa study (see [24] for information on the name algorithm). Therefore, the sample for the present study will be drawn from a data pool without positive name algorithms. Accordingly, no women with a Turkish migration background will be present in our sample, and this factor will be considered in the analysis and interpretation of the results.

### Decision aid

The DA was developed and piloted as part of this study. The DA and questionnaires for evaluation were programmed in Unipark, an online survey tool (QuestBack GmbH, Cologne, Germany). The structure of the DA was based on that of Mathieu et al. [19]. The DA was conceptualized as an interactive online tool consisting of a static information part and an interactive part.

We conducted a pilot study in March 2014, which showed that our study methods were feasible and acceptable to women aged 50. This quantitative pilot, to which 300 women were invited of which 53 participated, also showed that women accessed the DA and worked through it and that the questionnaires were accessed and answered. The pilot indicated that about a third of women dropped-out of the study prematurely. In a preceding qualitative pre-test both DA and questionnaires were evaluated with women of the target group through think-aloud protocols. The DA was additionally qualitatively piloted with experts from the National Network Women and Health.

In line with IPDAS criteria [25], the DA presents the options of choice (participation or non-participation in the MSP) in a decision-relevant context. The advantages and disadvantages of the MSP and their probabilities are described. These include the probabilities of a positive and negative screening result in absolute numbers supported by graphic presentations to allow women to form a realistic expectation regarding the initial outcome of the screening. We explain overdiagnosis, overtreatment, and the procedure following a positive result. Positive and negative information are presented in a balanced way according to current evidence. Step-by-step navigation through the websites of the interactive online DA facilitates a structured progress. Participants receive tailored feedback based on previous entries directly before making the decision. The studies from which the data for the DA originate are cited and provide the participants with easy access to the original sources via links.

A structured decision-making process in several steps is at the core of the DA. Participants have the opportunity to express personal values that are relevant for the decision and to clarify these for themselves. Participants have the option of printing their personal results and the static information section at the end [see Additional file 1] and to discuss matters with others. There is also an input window for remaining questions at the end of the DA.

#### Information part

The information part of the DA contains the standard information that women receive together with the invitation to the MSP [22]. The DA specifies all event probabilities based on the same population of 200 women over 20 years. In the presentation of outcome probabilities, the information part goes beyond the standard information. All probabilities are additionally represented as crowd-figure pictograms [see Additional file 1], thereby simultaneously indicating the positive and negative frames of outcomes. This type of pictorial information is beneficial [26]. Additionally, the probability of dying from breast cancer is presented in relation to the likelihood of all-cause mortality. Uncertainty in the evidence is described.

#### Interactive part

The interactive part consists of three steps. In the first step, the women assign the information items to the following categories: (1) in favour of mammography screening; (2) neither for nor against such screening; and (3) against the screening. In the second step, the women rate each point according to its importance in the decision. In the third step, the women make a decision. Through this approach, the importance of a personal value-based assessment of information is highlighted. An additional PDF file shows this in greater detail [see Additional file 1].

### Consent and data collection

The letter of invitation to the study, detailed study information, a consent form, and a return envelope will be sent by post 3 weeks in advance of the estimated date of receipt of the invitation to the MSP. The consent form will also provide for the possibility of specifying that the recipient does not have access to an email address. To increase the response rate, 1 week after the written invitation to participate in the study, a reminder and thank-you postcard will be mailed out.

Three weeks after the invitation is sent out, all women who gave informed consent and provided us with an email address will be emailed the link to the baseline questionnaire (T1). Women whose consent form is received 3-5 weeks after the invitation will receive the link at 5 weeks after the invitation. Women whose consent form is received later than 5 weeks after the invitation will receive the link at 7 weeks.

The link to the second assessment (T2) will be sent to participants 2 weeks after the first email. Women will be randomly assigned to the intervention or control group, and accordingly, receive a link either to the DA and second assessment or only to the second assessment. The link to the third assessment (T3) will be sent to the women 3 months after T2 when the screening appointment is assumed to have passed. A reminder and thank you will be emailed to all women who initially agreed to participate 10 days after sending the survey link at T1, T2, and T3.

### Primary outcome measure

The questionnaire was developed as part of another project (InEMa) and adapted for online use as well as for the evaluation of an intervention. For an overview of study outcome measures, see Table 1.

**Table 1:** Outcome measures

Measures	T1	T2	T3
Intention to participate in the MSP	X	X	
Uptake of the MSP			X
Attitude	X	X	X
Knowledge	X	X	X
Perceived behavioural control	X	X	
Subjective norms	X	X	
Decisional conflict	X	X	X
Decision regret			X
Result of the MSP			X
Result and acceptability of the DA		X	
Invitation status		X	
Decision stage	X		
Use of/experience with other screenings	X		
eHealth literacy	X		
Internet use	X		
Demographics	X		

*Informed choice* will be measured according to the three-dimensional classification framework of Marteau et al. [11], which covers knowledge, attitude, and implementation. The individual dimensions function additionally as secondary outcomes and are described below.

### Secondary outcome measures

*Intention to participate in the MSP* will be measured using one item regarding intention to participate in the next 3 months (yes/no/undecided).

*Uptake of the MSP* will be measured using one item regarding participation in the last 3 months (MSP/opportunistic screening/none).

*Attitude* will be measured using four items adapted from Marteau et al. [11] and according to the reasoned action approach of Fishbein and Ajzen [27]. Three semantic differentials (important/unimportant; a good thing/a bad thing; beneficial/harmful) will assess instrumental attitude. One semantic differential will assesses experiential attitude (pleasant/unpleasant). Items will be rated on a five-point scale.

*Knowledge* will be measured using seven multiple choice items, with two to four answer options. The questions will cover the following: (1) screening for people without symptoms; (2) frequency of positive screening results; (3) meaning of a positive result; (4) potential to detect every cancer; (5) more diagnoses with the MSP; (6) more breast cancer deaths without the MSP; and (7) overdiagnosis and overtreatment. For each item, “do not know” will be offered as an option. Most questions will ask for nun-numeric information. Only Question 2 will ask for numeric information through four ranges of values covering the base population used in the question. This information is considered to be important as all women participating in the MSP will receive a result and a realistic expectation on the probability of both positive and negative results is important.

*Perceived behavioural control* will be measured using two items developed following the reasoned action approach of Fishbein and Ajzen [27] and rated on a five-point scale (“Whether I participate in the MSP is up to me”; “If I wanted to, I could participate in the MSP”). Additionally, 15 items (four items at T2 and T3) measuring control beliefs will be rated on a five-point scale with the anchors of “agree” and “disagree”. The items will assess logistic barriers (e.g. not having time for the appointment), trust in the MSP, and fear of pain.

*Subjective norms* will be measured using two items developed according to the reasoned action approach of Fishbein and Ajzen [27] and rated on a five-point scale (“Most people who are important to me think that I should/should not participate in the MSP”; “Most people like me would/would not participate in the MSP”). Additionally, five items will measure normative beliefs rated on a five-point scale, ranging from “advise” to “disadvise” with the additional option of “no advice”. These items will assess the direction of advice of doctors, family, and friends.

*Decisional conflict* will be measured using the SURE (Sure of myself; Understand information; Risk-benefit ratio; Encouragement) test [28]. This four-item test with the answer options yes and no will assesses knowledge of options, clarity of importance of advantages and disadvantages, sufficient level of support, and being sure about the best choice. Since no German translation of this scale is available, the scale was translated as part of this study, and indices of reliability and validity will be published.

*Decision regret* will be measured using the Decision Regret Scale [29]. The five items will be rated on a five-point scale (“strongly agree” to “strongly disagree”). Since no German translation of this scale is available, the scale was translated as part of this study, and indices of reliability and validity will be published.

*Result of the MSP* will be assessed using two items regarding the result of the MSP and the result of further diagnostics. The answers will allow grouping into four categories: (1) negative result; (2) false-positive result; (3) true-positive result, and (4) positive result with further diagnostics pending.

*Result and acceptability of the DA* will be measured using one item assessing whether the tailored graphic in the DA was balanced, and four items will assess acceptability of the DA regarding length, amount of information (too much/too little/just right), one-sidedness (pro/balanced/against), and helpfulness in decision making (yes/neither/no).

*Invitation status* will be measured using two items assessing whether an invitation was received and whether the appointment specified in the invitation had already passed.

*Decision stage* will be measured using one item following IPDAS criteria with the answer options being “not thought about it”, “contemplating it”, “close to deciding”, and “choice already made”.

*Use of/experience with other screenings* will be measured using items on (1) the use of other screenings and (2) ever having received a false-positive screening result (including false-positive mammogram).

*eHealth literacy* will be measured using the eHealth Literacy Scale [30]. Its German translation has recently been validated [31].

*Internet use* will be measured using two items regarding time spent seeking information online and importance of the Internet for health information.

*Demographics* will be measured using items assessing education level, main language, health insurance, participation in a health insurance bonus programme, and breast cancer in a first-degree female relative.

### Sample size

Sample size was calculated using G* Power for the primary outcome (informed choice). Based on the study of Mathieu et al. [19], we determined a difference in the proportion of informed choices of 7 percentage points as the minimum clinically important difference for the power calculation. A sample size of 740 women (370 per arm based on a 1:1 allocation ratio) will enable the detection of a between-group difference of 7 percentage points given 80 % power and a 5 % (one-tailed) significance level. Allowing for an estimated response rate of 15 % and early drop-out of one-third of initial participants, we aim to invite 7400 women to take part in the study.

### Data analysis

The data will be exported from Unipark and analysed with SPSS version 21.0 (IBM Corp., Armonk, NY) and Mplus version 7.0 (Muthén & Muthén, Los Angeles, CA). Data cleaning and analyses will be performed using SPSS and Mplus syntax operations. We will conduct descriptive analyses to describe the study population. Possible baseline differences between trial arms will be statistically tested. We will adjust further analyses for imbalances in the baseline scores.

The primary analysis will be by intention to treat. As sensitivity analyses, only completers of all assessments will be analysed. The impact of the DA on the primary outcome will be analysed using *χ*^2^-tests. The numeric secondary outcomes will be analysed using analyses of variance; categorical secondary outcomes will be analysed using *χ*^2^-tests. Additionally, latent change model analyses will be performed for continuous longitudinal outcome data. Latent transition analyses will be performed for categorical longitudinal outcome data. To handle missing data, full information maximum likelihood estimation will be conducted in MPlus. To investigate the mediation and moderation effects of the secondary and demographic variables, mediation and moderation analyses will be performed using the PROCESS macro version 2.13 (Andrew F. Hayes, http://www.processmacro.org). in SPSS. As part of the questionnaire adaptation, factor, reliability, and correlation analyses will be performed.

## Discussion

This study will investigate the effects of a DA on informed choice in mammography screening. Studies on DAs in mammography screening are scarce. The DA developed for this study will consist of a static information part and an interactive part. No negative impact on the study participants is expected since the DA will include information that women ordinarily receive through usual care regardless of participation in this trial.

One strength of this study is its robust design and follow-up assessment at 3 months. This will allow evaluation of the long-term effects of the DA. Furthermore, in contrast to most randomized controlled trials, we will focus not only on the effectiveness of the DA, but also on mediating variables, thereby offering a model to explain the effect on informed choice.

There are also several challenges with this study. Especially, enrolling a sufficiently large sample is a major difficulty, which we hope to overcome by using reminders and underscoring the importance of this study and the future merits for women confronted with the MSP.

One potential disruptive influence on the study results will be previous screening experiences of participants. Therefore, only women aged 50 who are invited for the first time to the MSP will be included in the study. In addition, we will ask for information about previous experiences with breast cancer screening.

One potential problem with the online survey of a population sample is a low, selective participation rate. In the statistical analyses, we will adjust for interference by applying multivariate methods. No women with a Turkish migration background will be present in our sample, and this will have to be considered in the analysis.

In summary, this will be the first study to assess the impact of an online DA for the German MSP in a randomized controlled trial. The results of this research will be important for the following reasons: (1) they will provide information on the level of informed choice under usual care; (2) they will show whether a DA is helpful in increasing informed choice; and (3) they will indicate whether a DA is acceptable to women. The DA developed in this project will provide a support tool for decision making that can be used in further studies on informed choice and evaluated in other populations. If the DA proves to be effective in increasing the proportion of informed deciders, our results will be relevant to practice. Hence, the DA could be used on a large scale.

## Additional file

Additional file 1
**Sample personal decision aid PDF.** This PDF can be downloaded at the end of the online DA. It includes the static information part as well as the responses that were previously given by the user.

## Abbreviations

MSP: Mammography screening programme
DA: Decision aid
InEMa: Informed Choice of German and Turkish Women for Participation in the MSP (study title)
IPDAS: International Patient Decision Aids Standards
