# Supplementary material for: Does a decision aid improve informed choice in mammography screening? Study protocol for a randomized controlled trial
Source: BMC Womens Health. 2015 Jul 22;15:53. doi: 10.1186/s12905-015-0210-5 (PMC4510898; doi:10.1186/s12905-015-0210-5)
Supplement: Additional file 1 — Sample personal decision aid PDF. This PDF can be downloaded at the end of the online DA. It includes the static information part as well as the responses that were previously given by the user. [file 12905_2015_210_MOESM1_ESM.pdf]

Testung einer Entscheidungshilfe zum Mammographie-Screening-Programm

Eine Entscheidungshilfe für Frauen, die zum ersten Mal zum Mammographie-Screening-Programm eingeladen werden

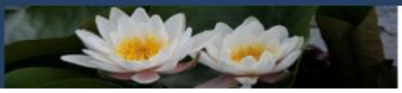

Soll ich am Mammographie-Screening-Programm teilnehmen?

Eine Entscheidungshilfe für Frauen im Alter von 50.

Warum soll ich mich entscheiden, ob ich am Mammographie-Screening-Programm teilnehme?
In Deutschland werden Frauen im Alter zwischen 50 und 69 alle zwei Jahre zum Mammographie-Screening-Programm eingeladen. Es gibt keine richtige oder falsche Antwort auf die Frage, ob jemand am Mammographie-Screening-Programm teilnehmen soll. **Es ist Ihre persönliche Entscheidung.**

Ist diese Entscheidungshilfe für mich?
Diese Entscheidungshilfe ist für Sie gedacht, wenn Sie:
- 50 Jahre alt sind; und
- momentan keine Brustbeschwerden haben.

Das Screening richtet sich an Frauen, die keine Beschwerden haben. Wenden Sie sich bei Veränderungen in Ihrer Brust immer direkt an eine Ärztin/einen Arzt. Mögliche Veränderungen sind Knoten, Dellen, Verhärtungen der Haut, sichtbare Verformungen, Einziehen der Brustwarze, Blutungen oder andere Absonderungen aus der Brustwarze.

Diese Entscheidungshilfe wurde von der Arbeitsgruppe Prävention und Gesundheitsförderung der Fakultät für Gesundheitswissenschaften der Universität Bielefeld entwickelt. Das Projekt wird durch die BARMER GEK gefördert.

Mehr Informationen zu den Quellen und dem Konzept finden Sie hier.
Quellen:
(1) Kooperationsgemeinschaft Mammographie, 2010. Informationen zum Mammographie-Screening: Programm zur Früherkennung von Brustkrebs für Frauen zwischen 50 und 69 Jahren.
(2) Nationales Netzwerk Frauen und Gesundheit in Kooperation mit der BARMER GEK, 2011. Brustkrebs Früherkennung - Informationen zur Mammografie - Eine Entscheidungshilfe.
Konzept:
(1) Richtlinien zur Erstellung von Entscheidungshilfen des Internationalen Netzwerks zur Erarbeitung von Standards für Patienten-Entscheidungshilfen (International Patient Decision Aid Standards Collaboration).
(2) Australische Entscheidungshilfe für Frauen im Alter von 40 der Universität Sydney.

Letzte Aktualisierung: März 2014
Bildquelle: Kamillo Kluth, Lizenz: CC-BY 2.0

Für Ihre Entscheidung ist es wichtig, dass Sie verstehen, welche Vor- und Nachteile das Screening hat.

Die folgenden Zahlen entsprechen den besten Schätzungen, können sich aber mit neuen Forschungsergebnissen ändern. Die Zahlen zum Mammographie-Screening wurden dem Merkblatt der Kooperationsgemeinschaft Mammographie "Informationen zum Mammographie-Screening: Programm zur Früherkennung von Brustkrebs für Frauen zwischen 50 und 69" entnommen.

Hier sind 200 Frauen abgebildet, die nicht am Screening teilnehmen (linke Seite) und 200 Frauen, die am Screening teilnehmen (rechte Seite). Die rosafarbigen Frauen stellen diejenigen dar, die aktuellen Studien zufolge innerhalb der nächsten 20 Jahre an Brustkrebs sterben werden. Die blauen Frauen stellen diejenigen dar, die innerhalb der nächsten 20 Jahre an anderen Todesursachen sterben werden.

**Kein Mammographie-Screening**<br><br>Wie viele Frauen im Alter von 50, die nicht am Mammographie-Screening teilnehmen, werden innerhalb der nächsten 20 Jahre an Brustkrebs sterben?	**10 Mammographie-Screenings (alle 2 Jahre)**<br>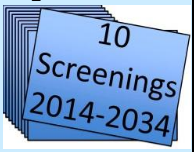<br>Wie viele Frauen im Alter von 50, die am Mammographie-Screening teilnehmen, werden innerhalb der nächsten 20 Jahre an Brustkrebs sterben?
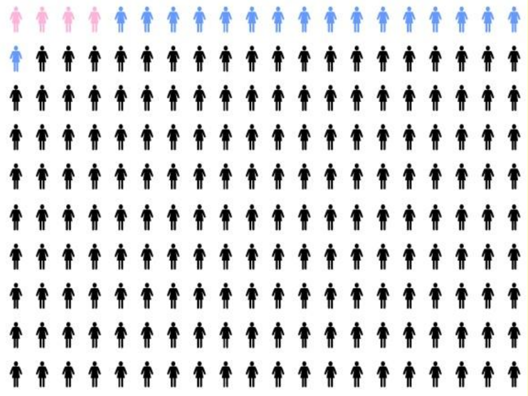	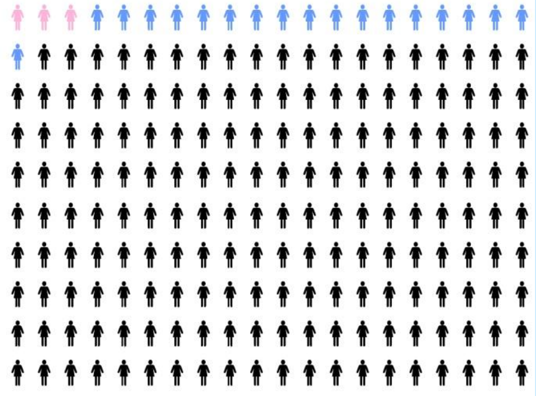
200 Frauen im Alter von 50 nehmen nicht am Mammographie-Screening teil.<br>**4 Frauen sterben** innerhalb der nächsten 20 Jahre **an Brustkrebs**.<br>**17 Frauen sterben** innerhalb der nächsten 20 Jahre **an anderen Todesursachen**.	200 Frauen im Alter von 50 nehmen alle zwei Jahre am Mammographie-Screening teil.<br>**3 Frauen sterben** innerhalb der nächsten 20 Jahre **an Brustkrebs**.<br>**18 Frauen sterben** innerhalb der nächsten 20 Jahre **an anderen Todesursachen**.

Wie bedeutsam ist Brustkrebs im Verhältnis zu anderen Todesursachen?
Wenn man **alle Todesursachen zusammen** betrachtet, **sterben** von 200 Frauen im Alter von 50 Jahren **21 Frauen** innerhalb der nächsten 20 Jahre - unabhängig davon, ob sie am Mammographie-Screening teilnehmen oder nicht.

10 Mammographie-Screenings (alle 2 Jahre)
Was passiert sonst noch mit den 200 Frauen im Alter von 50, die alle zwei Jahre innerhalb der nächsten 20 Jahre am Mammographie-Screening teilnehmen?

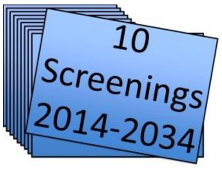

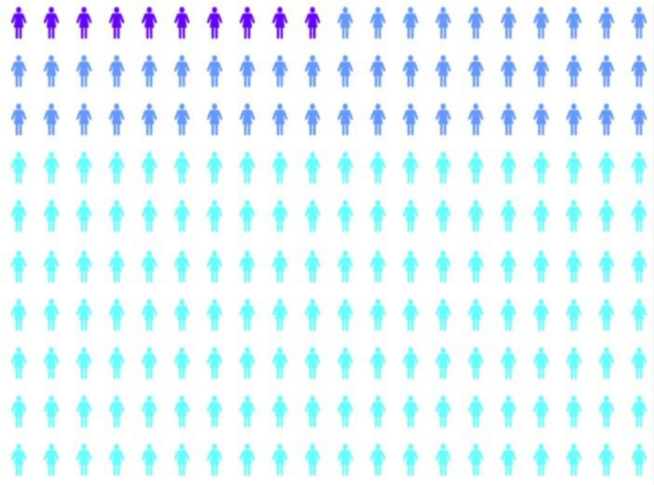

140 Frauen erhalten die **Rückmeldung**, dass sie **keinen Brustkrebs** haben.

50 Frauen haben eine auffällige Mammographie. Bei ihnen sind **weitere Tests** notwendig. Diese Tests zeigen jedoch, dass diese Frauen **keinen Brustkrebs** haben.

Bei **10 Frauen** wird der **Brustkrebs durch Screening entdeckt**. Eine von diesen 10 Frauen hätte ohne Mammographie zu Lebzeiten nichts von ihrem Brustkrebs bemerkt.

10 Mammographie-Screenings (alle 2 Jahre)
Was passiert sonst noch mit den 200 Frauen im Alter von 50, die alle zwei Jahre innerhalb der nächsten 20 Jahre am Mammographie-Screening teilnehmen?

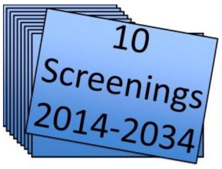

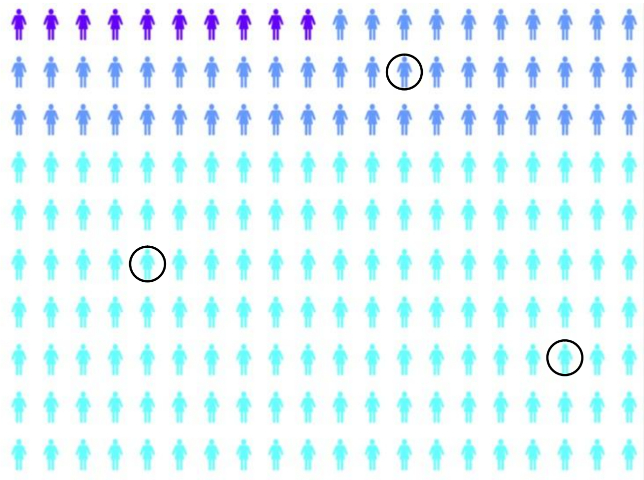

Zwischen den Mammographien erhalten **3 weitere Frauen** die Diagnose **Brustkrebs**, obwohl die Mammographie unauffällig war.

Es wird also bei **insgesamt 13 Frauen** innerhalb der nächsten 20 Jahre **Brustkrebs** diagnostiziert.

Die aktuellen Zahlen zum Mammographie-Screening kurz zusammengefasst

Was passiert mit 200 Frauen im Alter von 50, die alle zwei Jahre innerhalb der nächsten 20 Jahre am Mammographie-Screening teilnehmen?

- 1 Frau weniger stirbt an Brustkrebs.

- 1 Frau mehr erhält die Diagnose Brustkrebs, obwohl sie zu Lebzeiten nichts davon gemerkt hätte.

- Bei 50 Frauen werden nach einer auffälligen Mammographie weitere Tests durchgeführt, ohne dass Brustkrebs entdeckt wird. Sie könnten sich wegen dieses "Fehlalarms" Sorgen machen.

- 140 Frauen erhalten nach dem Screening die Rückmeldung, keinen Brustkrebs zu haben.

- 3 Frauen werden zwischen den Screenings mit Brustkrebs diagnostiziert, obwohl sie regelmäßig zum Mammographie-Screening gegangen sind.

Woran sollten Sie noch denken, bevor Sie sich entscheiden?

Für Ihre Entscheidung ist es auch wichtig zu wissen, wie das Mammographie-Screening-Programm funktioniert.

Wie funktioniert die Mammographie?

Die Mammographie ist eine Röntgenuntersuchung. Jede Brust wird zwischen zwei Plexiglasscheiben möglichst flach zusammengedrückt. Viele Frauen beschreiben die Untersuchung als unangenehm, manche als schmerzhaft, andere wiederum empfinden sie als unproblematisch.

Wie hoch ist die Strahlenbelastung durch die Mammographie?

Wie hoch die Belastung durch Röntgenstrahlen ist, kann derzeit nur geschätzt werden. Je jünger die Frau ist, desto strahlenempfindlicher ist ihr Brustgewebe. Mammographien dürfen deshalb zur Früherkennung erst bei Frauen ab 50 Jahren durchgeführt werden.

Ist Früherkennung Vorsorge?

Die Röntgenuntersuchung der Brust ist eine Momentaufnahme, keine Vorsorge:
Mit Hilfe der Mammographie kann ein Brusttumor möglicherweise in einem frühen Stadium erkannt werden. Die Mammographie kann aber keinen Brustkrebs verhindern.

Werden durch das Mammographie-Screening kleinere Tumore erkannt?

Ja, aber ob die Therapie weniger belastend ist, lässt sich nicht absehen. Chirurgische Eingriffe und Strahlenbehandlungen nehmen um etwa 30 Prozent zu
(Quelle: Gøtzsche, P.C.,& Jørgensen, K.J., 2013).

Werden durch das Screening mehr Frauen unnötig zur Brustkrebspatientin?

Durch die Früherkennung werden auch Veränderungen als Krebs oder als Krebsvorstufen diagnostiziert und behandelt, die ohne Mammographie nie auffällig und für die Frau auch nicht lebensbedrohlich geworden wären. Die Überdiagnosen von Brustkrebs führen zur Übertherapie, das heißt, es gibt zusätzliche Operationen, Strahlenbehandlungen und Chemotherapien, die es ohne Mammographie-Screening nicht gegeben hätte.
Eine frühere Diagnose führt nicht immer zu einer längeren Lebensdauer.

Ihre persönliche Entscheidungshilfe

Jede Frau kann frei entscheiden, ob sie am Mammographie-Screening teilnehmen möchte. Ihre persönliche Entscheidungshilfe soll Ihnen helfen, die Punkte abzuwägen, die für Ihre Entscheidung wichtig sind.

Im **ersten Schritt** haben Sie entschieden, was für Sie für und was gegen eine Teilnahme spricht.

Im **zweiten Schritt** haben Sie bewertet, wie wichtig die einzelnen Punkte für Ihre Entscheidung sind.

Im **dritten Schritt** haben Sie sich für oder gegen das Mammographie-Screening entschieden.

Auf den nächsten Seiten sehen Sie Ihre Ergebnisse noch einmal zusammengefasst.

Schritt 1 & 2

Sprechen diese Punkte für Sie für oder gegen eine Teilnahme am Mammographie-Screening?

Hier sind die Punkte, die für Sie für oder gegen eine Teilnahme am Mammographie-Screening gesprochen haben und deren Gewichtung noch einmal zusammengefasst.

**Für** das Mammographie-Screening sprechen für mich folgende Punkte:	**Gegen** das Mammographie-Screening sprechen für mich folgende Punkte:
	50 Frauen haben aufgrund des Mammographie-Screenings weitere Tests, bei denen sich der Verdacht auf Brustkrebs nicht bestätigt.
1 Frau stirbt aufgrund des Mammographie-Screenings nicht an Brustkrebs.	1 Frau erhält aufgrund des Mammographie-Screenings eine Brustkrebsdiagnose, obwohl sie zu Lebzeiten nichts davon bemerkt hätte.

Weitere Punkte:

(keine)

Schritt 3

Wie möchten Sie sich entscheiden?

So haben Sie sich entschieden:

Ich habe keine Entscheidung getroffen.

Welche Fragen sind für mich noch offen?

Mit diesen Fragen können Sie sich an Ihre Ärztin/Ihren Arzt oder an die Kooperationsgemeinschaft Mammographie wenden.

(keine)

Das Screening richtet sich an Frauen, die keine Beschwerden haben.
Wenden Sie sich bei Veränderungen in Ihrer Brust immer direkt an eine Ärztin/einen Arzt.
Mögliche Veränderungen sind Knoten, Dellen, Verhärtungen der Haut, sichtbare Verformungen, Einziehen der Brustwarze, Blutungen oder andere Absonderungen aus der Brustwarze.

Verwendete Literatur

Gøtzsche P.C., Jørgensen K.J., 2013. Screening for breast cancer with mammography. Cochrane Database of Systematic Reviews, 6. DOI: 10.1002/14651858.CD001877.pub5. http://summaries.cochrane.org/CD001877/screening-for-breast-cancer-with-mammography

Kooperationsgemeinschaft Mammographie, 2010. Informationen zum Mammographie- Screening: Programm zur Früherkennung von Brustkrebs für Frauen zwischen 50 und 69 Jahren. http://www.mammo-programm.de/cms_upload/datenpool/merkblatt_deutsch_web.pdf.

Nationales Netzwerk Frauen und Gesundheit in Kooperation mit der BARMER GEK, 2011. Brustkrebs Früherkennung - Informationen zur Mammografie - Eine Entscheidungshilfe. http://www.barmer-gek.de/barmer/web/Portale/Versicherte/Rundum-gutversichert/Infothek/Broschueren__und__Downloads/PDFs__Bilder__Broschueren__und__Downloads/Downloads/broschueren/ALLE__broschueren__neue_20Kategorien/Brustkrebs_20Fr_C3_BCherkennung,property=Data.pdf
